# Deep learning cone‐beam computed tomography image segmentation for the 3D visualization of mandibular infraosseous periodontal defects

**DOI:** 10.1002/jper.70058

**Published:** 2026-04-29

**Authors:** Daniel Palkovics, Balint Molnar, Csaba Pinter, David García‐Mato, Andres Diaz‐Pinto, Attila Tanacs, Andrea Dobos, Peter Windisch, Christoph A. Ramseier

**Affiliations:** ^1^ Department of Periodontology Semmelweis University Budapest Hungary; ^2^ Department of Periodontology University of Bern Bern Switzerland; ^3^ Dent.AI Medical Imaging Ltd. Budapest Hungary; ^4^ Empresa de Base Technológica Internacional de Canarias S.L. (EBATINCA) Las Palmas De Gran Canaria Spain; ^5^ School of Biomedical Engineering & Imaging Sciences King's College London St. Thomas' Campus St. Thomas' Hospital London UK; ^6^ Department of Image Processing and Computer Graphics University of Szeged Szeged Hungary; ^7^ Section of Periodontology Faculty of Odontology University Complutense of Madrid Madrid Spain

**Keywords:** 3D visualization, artificial intelligence, cone‐beam computed tomography, deep learning, infraosseous defects, periodontal defects, segmentation

## Abstract

**Background:**

The accurate assessment of infraosseous periodontal defects is crucial for effective diagnosis and treatment planning. Cone‐beam computed tomography (CBCT) enables detailed imaging of these defects; however, to leverage their full potential, CBCT images must be reconstructed in 3 dimensions (3D). Manual and semi‐automatic (SA) segmentation methods are time‐consuming and prone to human error. This study aimed to evaluate the performance of a deep learning (DL) model in segmenting mandibular infraosseous periodontal defects on CBCT scans.

**Methods:**

A multi‐stage Segmentation Residual Network (SegResNet)‐based DL model was used to segment CBCT scans from patients with stages III to IV periodontitis. Linear and volumetric measurements of infraosseous defects from DL‐generated 3D models were compared to those obtained using SA segmentation. The depth (INFRA), width (WIDTH), angle (ANGLE), and volume of 48 infraosseous defects were assessed on both DL and SA segmentations.

**Results:**

Measurements made on the DL and SA segmentations correlated strongly. The intraclass correlation coefficient (ICC) was 0.941 (*p* < 0.0001) for INFRA, 0.943 (*p* < 0.0001) for WIDTH, 0.889 (*p* < 0.0001) for ANGLE, and 0.948 (*p* < 0.0001) for defect volume. These results indicate high reliability of the DL model in capturing key characteristics of infraosseous periodontal defects.

**Conclusions:**

These findings support the use of DL‐based CBCT segmentation as a valuable tool for enhancing periodontal diagnosis. However, as this study was limited to mandibular defects, applicability to maxillary cases remains to be validated.

## Plain language summary

Periodontitis can cause severe bone loss around teeth, leading to the formation of complex defects that are challenging to diagnose and to treat. Cone‐beam computed tomography (CBCT) provides detailed 3D imaging of these defects, but current methods for segmenting CBCT scans and acquiring 3D models are time‐consuming and prone to human error. This study evaluated the ability of artificial intelligence (AI) to automatically segment infraosseous periodontal defects on CBCT images.

Using a SegResNet‐based deep learning model, the manuscript compared AI‐generated 3D models to the results of traditional semi‐automatic segmentation methods. The depth, width, angle, and volume of 48 infraosseous defects were measured, assessing whether AI could match human accuracy. The AI model performed exceptionally well, with strong statistical agreement between AI and human‐generated measurements.

By improving the way periodontal defects are visualized and measured, AI‐powered CBCT analysis could help dentists make better treatment decisions, reduce variability in diagnosis, and reduce the complication rates.

## 1 INTRODUCTION

The most recent European Federation of Periodontology (EFP) consensus report^1^ emphasized the growing integration of artificial intelligence (AI) ‐based systems into periodontology, enabling more consistent data‐driven diagnostics, screening, monitoring, and prognostic assessment. Previous studies have shown that AI can accurately detect and classify periodontitis on 2D radiographs, while supervised machine learning models have shown promising performance in predicting disease outcomes compared with conventional risk assessment methods. In terms of 3D data processing, the PerioAI concept presented by Tan et al. (2025)^2^ integrated cone‐beam computed tomography (CBCT) image segmentation, intraoral scans, and digital probing measurements within an AI‐based system to enhance periodontal diagnostics. Similarly, Kurt‐Bayrakdar et al. (2025)^3^ developed a deep learning (DL) ‐based system capable of detecting, segmenting, and classifying periodontal bone defects on CBCT images. These advances emphasize the potential of AI to complement conventional diagnostic approaches by providing detailed assessments of periodontal structures, thereby supporting treatment planning of regenerative therapy.

Clinical decision‐making and treatment planning processes in regenerative periodontal therapy are well‐established and backed by ample scientific evidence.^4^, ^5^ Among various patient and site‐specific factors, infraosseous defect morphology is one of the key elements determining both the appropriate regenerative surgical treatment and the anticipated treatment outcomes.^6^, ^7^, ^8^ Due to their more favorable configuration, in cases of containing (trench‐type) defects,^9^ minimal flap elevation and the sole use of biological mediators, such as enamel matrix derivatives^10^, ^11^ or platelet‐rich fibrin,^12^, ^13^ were demonstrated to result in successful outcomes. In contrast, the treatment of non‐containing (dehiscence‐type) defects^9^ requires an extended flap elevation and the application of graft materials or barrier membranes to achieve periodontal regeneration.^5^, ^14^, ^15^, ^16^ Furthermore, defect morphology significantly impacts regeneration potential, with 3‐ and 2‐wall defects demonstrating better outcomes compared to the limited regenerative potential of 1‐wall defects.^7^, ^8^, ^9^ For these reasons, in‐depth knowledge of infraosseous defect morphology is essential for appropriate treatment planning.

Clinical probing, periapical, and bitewing radiographs are the standard tools for periodontal diagnosis and treatment planning. However, their accuracy and reproducibility are limited by specific shortcomings. Clinical probing often shows low intra‐ and inter‐rater reliability due to factors such as variations in probing force, soft tissue edema, bleeding on probing, the presence of calculus, and tooth crowding.^17^, ^18^, ^19^ While conventional radiography offers an accessible and reproducible method for complementing clinical data by providing high‐resolution 2‐dimensional (2D) radiographic images, such radiographs cannot capture 3‐dimensional (3D) images, challenging the accurate assessment of complex infraosseous defect morphologies. On 2D radiographic images, overlapping anatomical structures may cover essential details, limiting their diagnostic capabilities. Multiple in vitro and in vivo studies have shown that, despite its lower overall resolution, CBCT outperforms conventional radiography in identifying specific periodontal defects, including furcation defects, 3‐wall intrabony defects, intrabony defects extending to the midbuccal/midlingual aspects of teeth (dehiscence‐type defects), and inter‐root craters.^20^, ^21^, ^22^, ^23^, ^24^ Nevertheless, the American Academy of Periodontology's 2017 consensus statement^25^, ^26^ did not endorse the routine clinical use of CBCT imaging in periodontal practice. CBCT scans may be justified on a case‐by‐case basis, provided the As Low As Diagnostically Acceptable, Indication‐oriented, and Patient‐specific (ALADAIP) principles^27^ are followed. To maximize the diagnostic value of CBCT, it is essential to fully exploit its 3D capabilities by obtaining 3D virtual models of the periodontal bone topography, enabling a more precise assessment and treatment plan. With semi‐automatic (SA) CBCT image segmentation methods, detailed 3D virtual models of teeth and the alveolar bone can be acquired.^24^ However, SA segmentation is limited by its time‐consuming nature and inconsistencies that arise from human errors in segmentation. The application of automatic DL‐based image segmentation methods can address these limitations. DL methods have been previously shown to produce accurate segmentations and 3D virtual models of dentoalveolar structures on CBCT scans. DL methods utilizing different convolutional neural network (CNN) architectures (e.g., 3D U‐Net,^28^ V‐Net,^29^ and SegResNet^30^) have previously achieved 92%–96% accuracy compared to SA segmentation.^31^, ^32^, ^33^

In our previous evaluation,^34^ a deep learning model based on a Segmentation Residual Network (SegResNet) architecture demonstrated reliable performance in segmenting periodontal bone topography. However, its performance in segmenting infraosseous periodontal lesions was not assessed separately. While such models may accurately delineate periodontal bone, they can still struggle to capture infraosseous defects due to substantial variability in their morphology, anatomical location, and extent.^35^, ^36^ In order to compensate for the diversity of infraosseous defects, a large sample training database must be established to improve the DL model's accuracy and robustness. Traditionally, segmentation accuracy is assessed using metrics that compare the overall similarity between the DL output and the reference SA segmentation. However, these global metrics do not capture how accurately the DL model performs in specific anatomical regions, such as the periodontal bone surrounding the teeth. To address this limitation, our previous study evaluated segmentation accuracy in the periodontal region using linear measurements between well‐defined anatomical landmarks, specifically the marginal bone and the cementoenamel junction. Comparing linear measurements allows for the assessment of segmentation accuracy in clinically relevant areas and offers clinicians more easily interpretable information.

Hence, this investigation aimed to evaluate the accuracy of a DL CBCT segmentation model for the segmentation of infraosseous periodontal defects, by comparing linear and volumetric measurements of such defects made on 3D models acquired by either DL or SA segmentation.

## 2 MATERIALS AND METHODS

### 2.1 Study design

The current study aimed to evaluate the performance of a previously introduced multi‐stage SegResNet‐based DL model^30^, ^33^, ^34^ for the segmentation of infraosseous periodontal defects visible on CBCT scans. The SegResNet CNN, similar to a 3D U‐Net^28^ has an encoder‐decoder‐based structure, but with an asymmetrically larger encoder portion for feature extraction. CBCT scans used for training, testing, and validation were derived from patients diagnosed with stages III–IV periodontitis undergoing periodontal rehabilitation at the Department of Periodontology, Semmelweis University, Budapest. The model was trained on 70 CBCT scans and evaluated on an independent test dataset of 21 CBCTs. The test dataset size was selected to meet the sample size requirement for infraosseous defects. In the present study, the performance of the SegResNet deep learning model in segmenting infraosseous periodontal defects was evaluated using both linear and volumetric measurements. Depth, width, angle, and volume of 48 infraosseous defects were assessed in the current study. Prior to inclusion, all CBCT data were anonymized. The CBCT scans utilized for training, validation, and testing were sourced from routine clinical practice and were originally acquired for purposes unrelated to periodontal treatment planning. All patients provided written informed consent prior to participation. This study was approved by the human subjects ethics board of Semmelweis University's Regional Institutional Scientific and Research Ethics Committee (approval number SE RKEB 138/2023) and was conducted per the Helsinki Declaration of 1975, as revised in 2013.^37^ The study followed the basic guidelines of Schwendicke et al.^38^ (2021) and the Checklist for Artificial Intelligence in Medical Imaging (CLAIM) guidelines.^39^

### 2.2 Model architecture and performance

The architecture and training strategy of the multi‐stage SegResNet‐based deep learning model have been described in detail elsewhere.^34^ Briefly, Stage 1 performed classification and segmentation of teeth, and Stage 2 performed mandible segmentation. The encoder comprises ResNet blocks with 2 convolutional layers, group normalization, and ReLU activation, using 3 × 3 × 3 convolutions with an initial filter size of 32. The decoder mirrors the encoder but with a single block per spatial level, halving feature numbers and doubling spatial dimensions at each step, with skip connections from the encoder. A variational autoencoder (VAE)^40^ branch at the encoder endpoint was added to enhance feature clustering and regularization. The model was developed using MONAI (https://github.com/Project‐MONAI/MONAI), an open‐source PyTorch framework. Compared to SA segmentation, the current DL model achieved a mean DSC of 0.9650 ± 0.0097, an IoU of 0.9340 ± 0.0180, and an HD95 of 0.4820 mm ± 0.1269 mm, indicating strong agreement.^34^

### 2.3 Data

Inclusion criteria:

- CBCT scans of partially edentulous patients diagnosed with stages III–IV periodontitis
- CBCT scans with restorations and root canal‐treated teeth
- CBCT scans with titanium pins from previous reconstructive surgeries
- CBCT scans containing the entire maxillofacial region

Although objects, such as root canal fillings and restorations, may introduce imaging artifacts and occasionally may alter the definition of periodontal bone defects, the inclusion of such images was essential to ensure the model's clinical relevance.

Exclusion criteria:

- Conventional computed tomography scans of the head and neck region
- CBCT scans of fully dentate patients with no visible hard tissue defects
- CBCT scans with dental implants
- CBCT scans of fully edentulous patients

Dental implants were excluded because the current AI model was not trained to recognize or segment implant structures, and their inclusion could confound defect detection in their close vicinity.

Image acquisition parameters of the CBCT scans selected for testing in the current article are as follows:

- Planmeca Viso (Planmeca, Helsinki, Finland; *n* = 8)
  - voxel size: 150 µm
  - voltage: 100 kV
  - tube current: 12 mA
  - field‐of‐view: 14 × 10 cm
- Planmeca Promax 3D (Planmeca, Helsinki, Finland; *n* = 4)
  - voxel size: 150 µm
  - voltage: 90 kV
  - tube current: 10 mA
  - field‐of‐view: 16 × 10 cm
- KODAK CS 8100 Evo (Carestream Health, Rochester, NY, USA; *n* = 5)
  - voxel size: 150 µm
  - voltage: 90 kV
  - tube current: 2 mA
  - field‐of‐view: 8 × 10 cm
- I‐CAT FLX (KaVo Dental GmbH, Bieberach an de Riß, Germany; *n* = 4)
  - voxel size: 300 µm
  - voltage: 120 kV
  - tube current: 36 mA
  - field‐of‐view: 16 × 10 cm

### 2.4 Testing

#### 2.4.1 Acquisition of reference SA segmentations

SA segmentations were performed using an open‐source medical image processing software (3D Slicer,^41^, ^42^ https://www.slicer.org). SA segmentation was performed using a sequence of region‐based segmentation methods within the toolset of the *“Segment editor”* module of 3D Slicer.^42^ The mandible was segmented with a region‐growing^43^, ^44^ method, while the teeth were segmented using the watershed^45^ technique.^33^ The mandibular canal was segmented using the “Draw tube” effect. Manual segmentation tools were used to correct occasional errors.^46^ SA segmentations were performed by a single rater (D.P.) with 6 years of experience in medical image processing. The segmentation results were carefully inspected by a clinician with more than 30 years of clinical experience (P.W.).

#### 2.4.2 Intra‐rater reliability

Intra‐rater reliability (IRR) was evaluated to confirm the consistency and accuracy of the SA segmentations of CBCT scans and linear measurements using the intraclass correlation coefficient (ICC). A strong positive correlation was observed between the volumes of SA segmentations, with an ICC of 0.9998 (*p* < 0.0001). The ICC for linear measurements was found to be 0.9993 (*p* < 0.0001), indicating similarly excellent consistency.

#### 2.4.3 Linear measurements of infraosseous defect characteristics

Linear periodontal measurements were performed at the infraosseous defects to assess the depth, width, and angle of the infrabony component. For each tooth, the CBCT dataset was reoriented to align with its corono‐apical axis—defined as the line connecting the radiographic apex to the center of the crown—thereby matching the angulation, inclination, and rotation of each tooth. This ensured that all measurements were taken in a standardized anatomical orientation. All aforementioned characteristics were assessed at 3 aspects (buccal, middle, and lingual) of each infraosseous defect.

**FIGURE 1:**
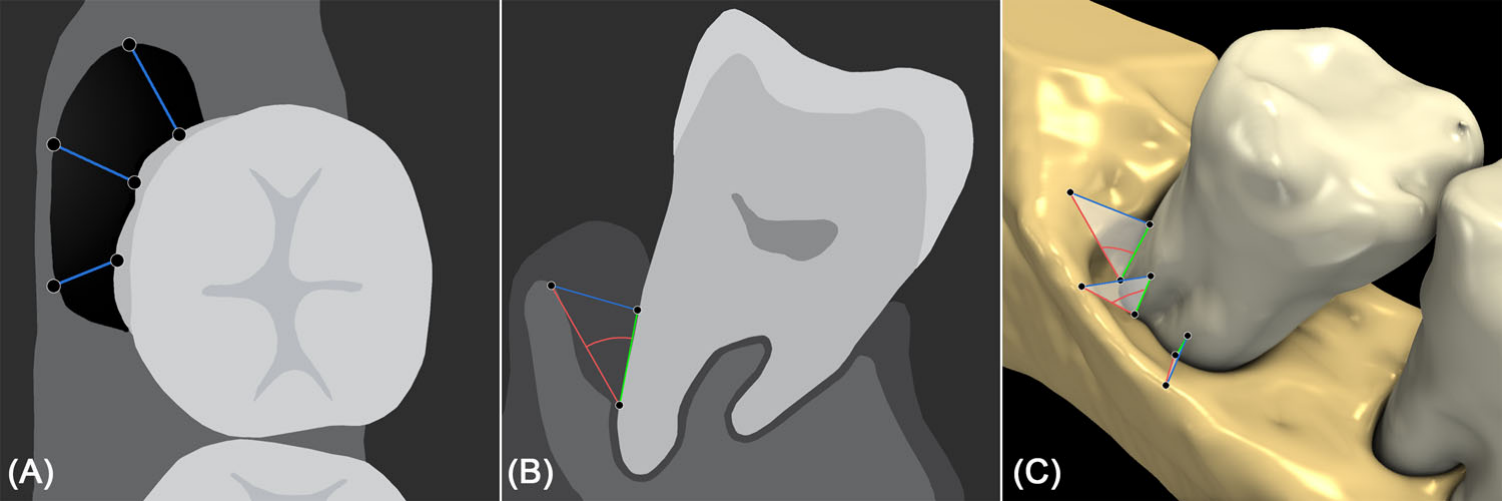
Linear measurements of infraosseous defect characteristics. (A) Three measurements were made at each infraosseous defect. The schematic displays a lingually located defect with the 3 sites being mesial, middle, and distal. However, most defects were located interdentally, in which case the measurement sites were buccal, middle, and lingual. (B) The vertical dimension of the intrabony component was measured parallel to the tooth surface from the level of the marginal bone crest and the bottom of the defect (INTRA). Horizontal dimensions of the infraosseous defects were measured perpendicular to the tooth surface between the marginal bone crest and the tooth surface (WIDTH). The angulation of the defect was indicated by the angle between the tooth surface and the bony wall of the infraosseous defect. (C) 3D demonstration of the measurements. Axial, coronal, and sagittal planes were rotated to match the inclination, angulation, and rotation of each tooth, respectively.

The primary outcome of this study was to assess the agreement in infrabony defect depth measurements between the SA and DL segmentations. The depth of the infrabony defect was defined as the vertical distance between the marginal bone crest and the bottom of the defect (INFRA). The width of the defect measured at the same aspect was defined as the horizontal distance from the most coronal points of the bone crest to the root surface (WIDTH). The angle of the defect was defined as that between the bony wall of the infrabony component and the associated tooth surface (ANGLE). All 3 parameters were measured on 3D models generated by both DL and SA segmentation; thereafter, the values were compared to assess the precision of the SegResNet‐based DL model in the segmentation of infraosseous periodontal defects (Figure 1). Linear measurements were performed using tools in the “Markups” module of 3D Slicer.

#### 2.4.4 Volumetric measurements of infraosseous defects

**FIGURE 2:**
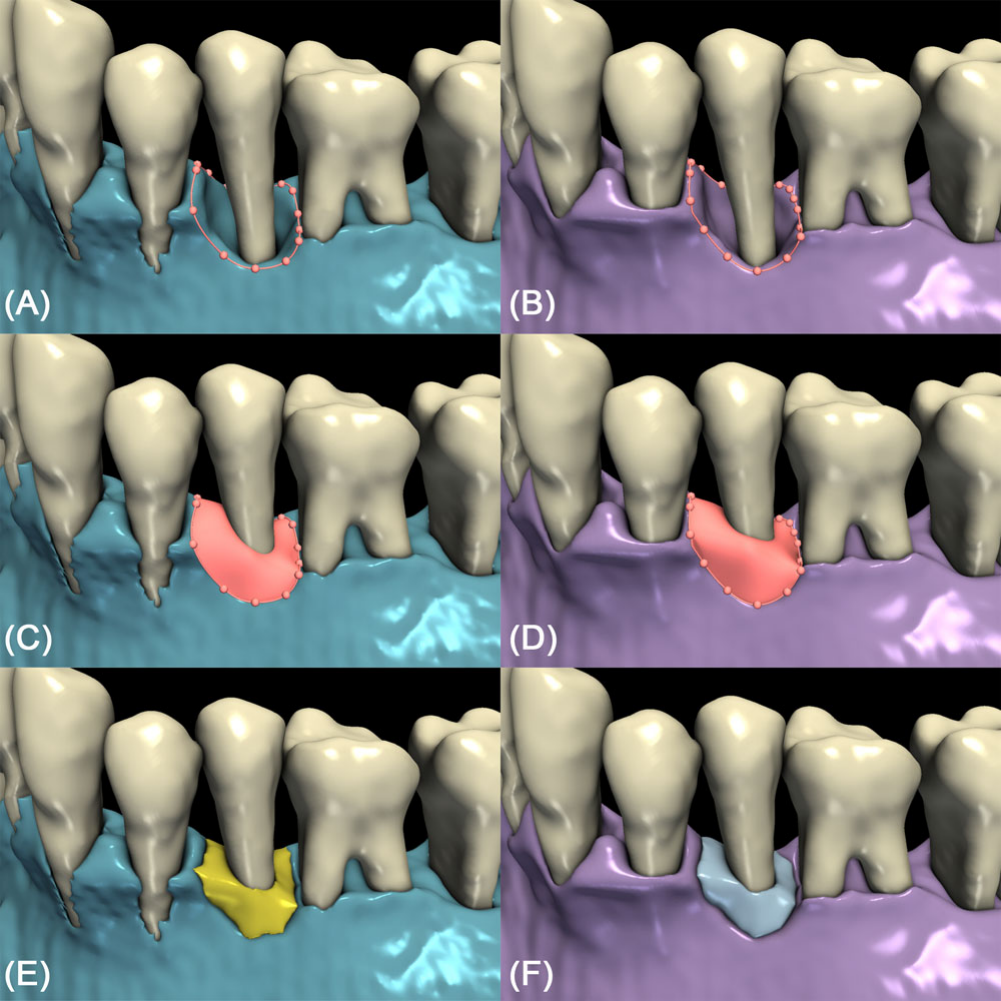
Comparison of infraosseous defect volumes on the deep learning (DL) and semi‐automatic (SA) segmentations. (A, B) Closed curve markup manually drawn on the marginal bone crest around the defect‐associated tooth; (A) DL segmentation, (B) SA segmentation. (C, D) A flat surface model was automatically generated over the orifice of the alveolar socket using the “Baffle planner” module of 3D Slicer, marking the crestal‐most point of the infrabony component. (E, F) The space below this flat surface model was segmented using manual tools to acquire segmentations for the infraosseous defects.

In addition to linear measurements, the volumes of the infraosseous defects were also measured and compared between the DL and SA segmentations using a methodology described in an earlier publication.^47^ Initially, a closed curve markup was drawn manually on the marginal bone crest around the defect‐associated tooth. Thereafter, a flat surface model was automatically generated over the orifice of the alveolar socket based on the input curve using the *“Baffle planner (Cardiac extension)”* ^48^ module of 3D Slicer. This flat surface model marked the crestal‐most point of the infrabony component of the defect. The space below this flat surface model was segmented using manual tools of the *“Segment editor”* module to acquire segmentations for the infraosseous defects. These steps were replicated on both the DL and SA segmentations. Subsequently, the volumes of the infraosseous defect segmentations were calculated in cubic millimeters (mm^3^) using the *“Segment statistics”* module of 3D Slicer (Figure 2).

### 2.5 Statistical analysis

The sample size calculation was based on our preliminary data^49^ comparing the INTRA values of infraosseous defects from SA and DL segmentations. In this evaluation, the mean difference between the 2 methods was 0.150 mm, with a standard deviation of 0.410 mm. Using a significance level (α) of 0.05, a statistical power (1−β) of 0.80, and an effect size of 0.366, it was determined that the evaluation of 48 infraossseous defects would be required to achieve adequate statistical power. Sample size calculation was performed using G*Power 3.1.9.7 (Heinrich Heine University, Düsseldorf, Germany).^50^

Descriptive statistics were calculated for all variables, with data presented as mean ± standard deviation. The Shapiro–Wilk test was used to assess the normality of variable distributions. Correlations between DL and SA measurements were evaluated using Pearson's correlation (Pearson's *r*) if data were normally distributed and Spearman's rank correlation (Spearman's *ρ*) for non‐normally distributed data. Additionally, the ICC was calculated to determine the correlation between the DL and SA measurements. Bland–Altman plots were constructed to assess the agreement and bias between measurements obtained from the DL and SA segmentations. Statistical significance was set at *p* < 0.05 for all analyses. All statistical analyses were performed using the Stata statistical software package (version 18; StataCorp LLC, College Station, TX, USA).

## 3 RESULTS

### 3.1 Infraosseous defect demographics

A total of 48 infraosseous defects identified across 21 CBCT scans were evaluated to test the DL model's performance. Of these, 5 defects were associated with incisors, 4 with canines, 21 with premolars, and 18 with molars. Except for 1 defect located on the lingual surface of a molar, all defects were interproximal. Most defects were combined defects with varying numbers of residual walls in the vertical dimension and were classified as either narrow‐angle (< 25°), medium‐angle (25–37°), or wide‐angle (> 37°) defects. A detailed description of the individual defect morphologies is provided in Table 1.

**TABLE 1:** Defect demographics.

Ref. no.^a^	Defect no.	Location	No. of walls^b^	Angle^c^
PAT_1	1	45 distal	3‐wall	Wide
PAT_2	2	44 distal	2‐wall	Medium
PAT_3	3	34 mesial	2‐3‐wall	Wide
PAT_4	4	31 mesial	2‐wall	Medium
	5	36 mesial	2‐3‐wall	Wide
	6	37 distal	3‐wall	Wide
PAT_5	7	34 mesial	2‐3‐wall	Narrow
	8	35 distal	2‐3‐wall	Wide
	9	47 distal	1‐2‐3‐wall	Wide
PAT_6	10	43 mesial	2‐wall	Wide
	11	44 mesial^d^	4‐wall	Narrow
	12	44 distal^d^	4‐wall	Medium
PAT_7	13	45 mesial	1‐wall	Wide
PAT_8	14	34 mesial	3‐wall	Wide
	15	37 lingual	2‐3‐wall	Wide
PAT_9	16	44 mesial	2‐3‐wall	Wide
	17	37 mesial	1‐wall	Wide
PAT_10	18	44 distal	2‐wall	Medium
	19	32 distal	2‐3‐wall	Medium
	20	36 mesial	2‐3‐wall	Medium
	21	36 distal	1‐2‐wall	Medium
PAT_11	22	45 distal	1‐2‐3‐wall	Medium
PAT_12	23	34 distal	2‐3‐wall	Wide
	24	36 mesial	2‐3‐wall	Wide
	25	36 distal	2‐wall	Wide
PAT_13	26	44 lingual	4‐wall	Medium
PAT_14	27	37 mesial	2‐3‐wall	Wide
	28	46 mesial	3‐wall	Medium
PAT_15	29	44 mesial	2‐wall	Narrow
	30	45 mesial	1‐wall	Wide
	31	47 mesial	3‐wall	Medium
PAT_16	32	44 distal	2‐3‐wall	Medium
	33	45 distal	3‐wall	Wide
	34	32 distal	1‐wall	Medium
PAT_17	35	46 mesial	3‐wall	Wide
	36	47 distal	1‐wall	Wide
PAT_18	37	33 distal	2‐3‐wall	Medium
	38	34 lingual	3‐wall	Wide
	39	37 mesial	2‐3‐wall	Narrow
	40	42 distal	2‐3‐wall	Medium
	41	44 distal	1‐wall	Medium
PAT_19	42	43 mesial	1‐2‐wall	Medium
	43	33 mesial	1‐2‐wall	Medium
	44	34 distal	2‐wall	Medium
PAT_20	45	47 distal	2‐3‐wall	Wide
	46	32 distal	1‐wall	Wide
PAT_21	47	37 mesial	2‐3‐wall	Narrow
	48	47 distal	3‐wall	Medium

^a^ CBCT reference number.

^b^ Number of bony walls: the first number always refers to the number of walls at the most coronal aspect.

^c^ Defect angle categorized according to Nibali & Cortellini.^9^.

^d^ Circumdental defect of PAT_6 tooth 44 with missing buccal wall.

### 3.2 Accuracy of linear periodontal measurements

#### 3.2.1 Depth of the infrabony component (INFRA)

**FIGURE 3:**
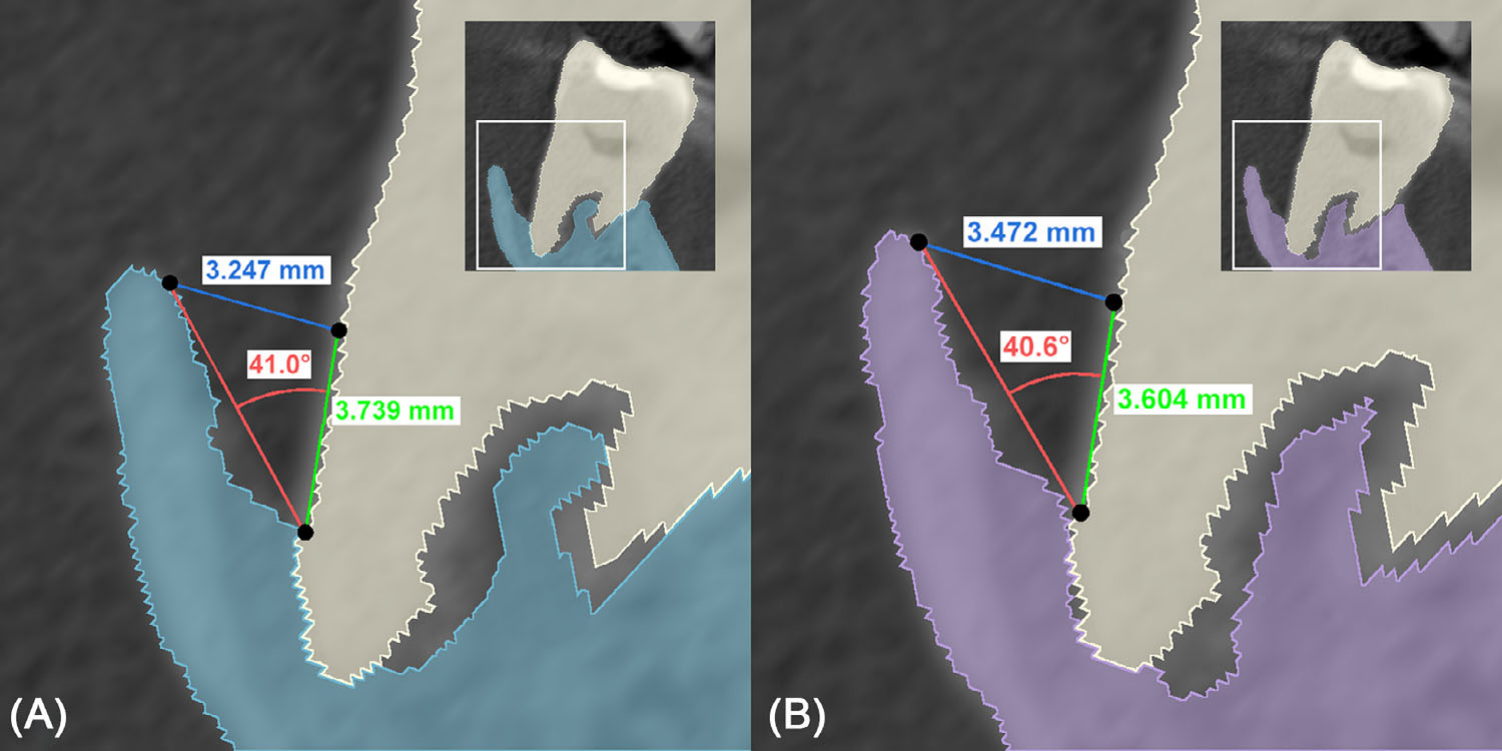
Linear measurements of infraosseous periodontal defects on 3D models generated by deep learning (DL) and semi‐automatic (SA) segmentations. Defect depth (INFRA, green) was defined as the vertical distance from the marginal crest to the base of the defect. Width of the intrabony component (WIDTH, blue) was measured as the horizontal distance from the crest to the root surface. The angle (ANGLE, red) was determined between the bony wall and the root surface. Measurements were standardized by reorienting each CBCT dataset to the corono‐apical axis and assessed at buccal, middle, and lingual aspects. (A) Linear measurements on the DL model. (B) Linear measurements on the SA model.

The mean overall INFRA values for all infraosseous defects across the 3 measurement sites were 3.618 mm ± 1.286 mm for DL segmentations and 3.849 mm ± 1.354 mm for SA segmentations. A strong and statistically significant correlation was detected between the 2 measurements (ICC: 0.941, *p* < 0.0001; Spearman's *ρ*: 0.941, *p* < 0.0001). INFRA values and statistical correlations are summarized in Table 2 and visualized in Figure 3.

**TABLE 2:** Linear measurements of infraosseous defect depth.

	INFRA (mm)		WIDTH (mm)		ANGLE (mm)		Volume (mm^3^)	
Parameter	DL^a^	SA^b^	DL	SA	DL	SA	DL	SA
Mean	3.618	3.849	2.881	3.421	37.568	36.406	47.946	57.231
St. dev.^c^	1.286	1.354	0.885	1.343	8.635	9.076	33.027	36.642
Minimum	1.089	1.345	1.142	1.958	14.400	12.300	9.244	11.711
Maximum	7.020	7.741	6.233	6.840	60.700	62.200	166.278	183.433
Median	3.397	3.725	2.742	3.036	37.300	35.950	39.289	2.881
ICC^d^	0.941 (*p* < 0.0001)		0.943 (*p* < 0.0001)		0.889 (*p* < 0.0001)		0.948 (*p* < 0.0001)	
Correlation	0.943^e^ (*p* < 0.0001)		0.953^e^ (*p* < 0.0001)		0.898^f^ (*p* < 0.0001)		0.966^e^ (*p* < 0.0001)	

^a^ Deep learning.

^b^ Semi‐automatic.

^c^ Standard deviation.

^d^ Intraclass correlation coefficient.

^e^ Spearman's *ρ*.

^f^ Pearson's *r*.

**FIGURE 4:**
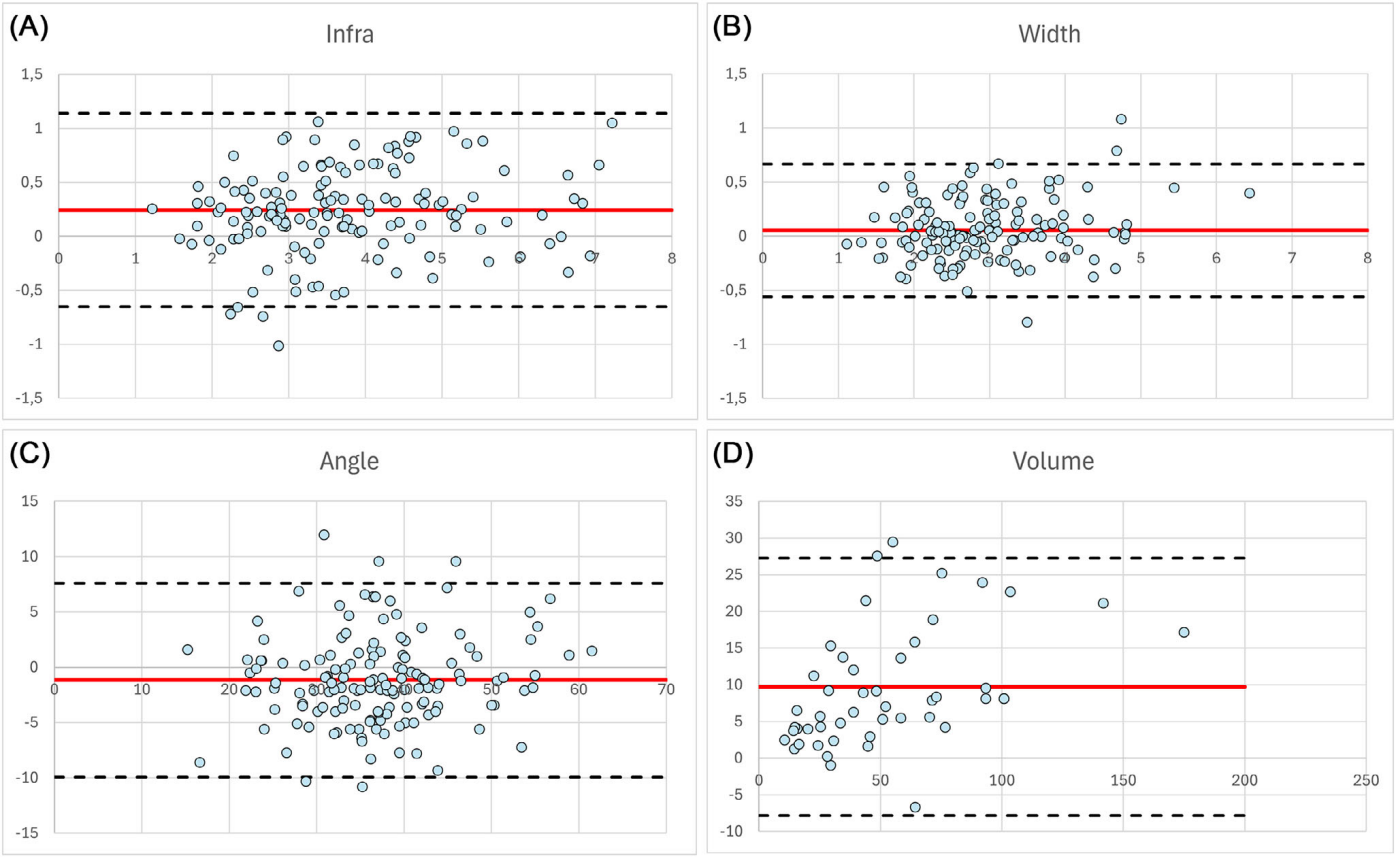
Bland–Altman plots comparing measurements of infraosseous periodontal defects obtained from deep learning (DL) and semi‐automatic (SA) segmentations. (A) Defect depth (INFRA) showed a small positive bias (0.241 mm) with narrow limits of agreement. (B) Defect width (WIDTH) demonstrated negligible bias (0.053 mm) and strong consistency. (C) Defect angle (ANGLE) showed minimal bias (–1.16°) with slightly wider variability. (D) Defect volume (VOLUME) exhibited a positive bias (9.7 mm^3^) and broader limits of agreement, reflecting systematic underestimation of defect size by DL segmentation to over‐segmentation of bone.

Bland–Altman analysis of INFRA values measured on DL and SA segmentations demonstrated a small positive bias of 0.241 mm, indicating a negligible systematic difference between measurements. Limits of agreement ranged from –0.655 mm to 1.138 mm, with the majority of values falling well within this interval. The differences were evenly distributed across the range of measurements. The Bland–Altman plot is visible in Figure 4A.

#### 3.2.2 Width of the infrabony component (WIDTH)

WIDTH values averaged 2.881 mm ± 0.885 mm on the DL segmentations and 2.943 mm ± 0.931 mm on the SA models. A strong statistical correlation was observed between the 2 measurement methods, with an ICC of 0.943 (*p* < 0.0001) and Spearman's *ρ* of 0.953 (*p* < 0.0001). WIDTH values and statistical correlations are summarized in Table 2 and visualized in Figure 3.

For the WIDTH measurements, the Bland–Altman analysis revealed a negligible mean bias of approximately 0.053 mm, suggesting virtually no systematic difference between DL and SA measurements. The 95% limits of agreement were narrow (–0.561 to 0.667), indicating strong consistency and minimal measurement error. Differences were evenly distributed across the range of measurement values, and only a few isolated outliers were observed. The Bland–Altman plot is visible in Figure 4B.

#### 3.2.3 Angle of the infrabony component (ANGLE)

The mean overall defect angle was 37.568° ± 8.635° and 36.406° ± 9.076° on the DL and the SA segmentations, respectively. The ANGLE measurements on the 2 segmentations were found to correlate strongly, with an ICC of 0.889 (*p* < 0.0001) and a Pearson's *r* of 0.898 (*p* < 0.0001). The ANGLE values and statistical correlations are summarized in Table 2 and visualized in Figure 3.

For the infraosseous defect angle measurements, Bland–Altman analysis indicated a negligible bias (–1.16°), suggesting essentially no systematic error between the 2 measurements. The limits of agreement ranged from –9.931 to 7.607, reflecting a slightly wider variability. Although the majority of data points clustered around the mean difference, several outliers were observed; however, no proportional bias could be detected. The Bland–Altman plot is visible in Figure 4C.

### 3.3 Volumetric defect measurements

The mean infraosseous defect volumes were 47.946 mm^3^ ± 33.027 mm^3^ on the DL segmentations and 57.7891 mm^3^ ± 36.642 mm^3^ on the SA segmentations. These results demonstrated high levels of statistical correlation, with the ICC being 0.948 (*p* < 0.0001) and Pearson's *r* being 0.966 (*p* < 0.0001). The volumetric measurement values are summarized in Table 2.

For volume measurements, Bland–Altman analysis revealed a positive systematic bias of 9.702 mm^3^, indicating that measurements on DL segmentations consistently underestimated defect volumes compared to SA segmentations. Compared to other variables, a relatively wide limit of agreement could be detected (from –7.858 mm^3^ to 27.263 mm^3^), reflecting a variability between measurements. While most values fell within the agreement range, several outliers and a tendency toward larger discrepancies at higher values were present, suggesting a possible proportional bias (Figure 4D).

## 4 DISCUSSION

The present study evaluated the accuracy of a SegResNet‐based DL CBCT segmentation model for infraosseous periodontal defects by comparing linear‐ and volumetric measurements between DL and SA segmentations. Our findings demonstrated that measurements performed on the DL model are generally accurate and comparable to those performed on SA segmentations. These findings suggest that the developed DL model provides adequate performance not only for the segmentation of mandibular periodontal bone topography in general,^34^ but also specifically for infraosseous periodontal defects. Unlike our prior publication, which focused on overall segmentation accuracy (DSC, IoU, HD 95) of the periodontal bone topography, the current study evaluated the model's accuracy of infraosseous periodontal defects through linear measurements. Furthermore, linear measurements may be easier for clinicians to interpret. CBCT linear measurements are shown to be highly accurate and reliable for periodontal measurements.^20^, ^21^, ^22^, ^23^

Despite the relatively small database that the utilized model was trained on, a relatively high accuracy could be achieved,^34^ DSC, IoU, and HD 95 values were comparable with data found in the literature on automatic mandible segmentation.^31^, ^32^ This performance may be attributed to the variability in the training dataset, and the integration of a VAE branch facilitated the learning of meaningful latent representations, thereby enhancing the model's ability to generalize to unseen examples.^40^ Trueness of DL‐based segmentation achieved with the SegResNet model in the current paper was found to be similar to those reported in other similar articles.^31^, ^32^, ^51^ Verhelst et al. (2021)^31^ reporting a mean DSC of 0.9722 utilizing a 3D U‐Net model for mandibular segmentation. Cui et al. (2022)^32^ trained a V‐Net‐based model on a large, heterogeneous dataset and achieved a mean DSC of 0.9450. Similarly, Liu et al. (2024)^51^ introduced a transformer‐based pipeline (Swin‐UNETR) for multi‐structure CBCT segmentation, reporting DSC values above 98% for mandibular structures. Among very few studies on this topic, the recent work of Kurt–Bayrakdar et al. (2025)^3^ have applied a multi‐stage nnU‐Net‐based framework, aiming to automatically segment and classify specific types of periodontal defects. The authors reported a DSC of 0.59 and an IoU of 0.42. The authors highlighted the difficulty of infraosseous defect segmentation and suggested focusing on preprocessing strategies to enhance segmentation accuracy. As segmentation accuracy was assessed using different methodologies, a direct comparison cannot be made. It should also be noted that, in contrast to the study by Kurt–Bayrakdar et al. (2025),^3^ the current analysis did not evaluate the model's performance for defect classification.

Nevertheless, the present study demonstrated consistently high correlations with ICC values between DL and SA measurements being 0.941 (INFRA), 0.943 (WIDTH), 0.889 (ANGLE), and 0.966 (volume). Bland–Altman analysis further confirmed the close agreement between DL‐ and SA‐based measurements. Some outliers were detected, most of which were associated with lower resolution (300 µm) I‐CAT CBCT scans. These results may indicate the model's limited applicability to lower‐resolution CBCT scans. For INFRA, WIDTH only minimal bias and narrow limits of agreement were observed, while ANGLE measurements showed slightly wider variability. Volume measurements, however, displayed a more pronounced positive bias and broader limits of agreement, reflecting that the DL model tended to over‐segment bone at infraosseous defects, thereby underestimating true defect volumes. Minor discrepancies in the vertical and horizontal dimensions are accumulated during volume calculation, resulting in a disproportionately larger volumetric difference. Additionally, wider infraosseous defects were segmented more effectively, likely due to the greater distance between the marginal bone and teeth (2 radiopaque structures), which may have enhanced the model's edge detection capabilities. Similarly, to our previous study,^34^ a discrepancy in the segmentation of thin buccal bone could also be observed. It should also be noted that the segmentation of thin buccal bone around teeth is challenging even for experts. This inaccuracy, however, was primarily observed on the buccal surface of teeth and was less pronounced in the interdental regions; nevertheless, it may still compromise the accuracy of 3D defect visualization and, in turn, adversely affect surgical treatment planning.

The main clinical relevance of the current approach lies in the improved visualization of 3D defect morphology, which provides practical advantages for surgical planning. The 3D assessment allows clinicians to determine the most convenient access to the defect. This facilitates the application of minimally invasive flap designs, thereby reducing surgical morbidity while enhancing regenerative potential. Moreover, the precise depiction of defect architecture allows a more individualized selection of regenerative strategies.

An inherent limitation of this study is that it did not evaluate the segmentation accuracy of furcation defects, one of the main indications for CBCT scans for periodontal diagnostic purposes. This was due to the low number of furcation‐involved teeth in the current sample, which was insufficient for statistical significance testing. However, based on our observations, it is reasonable to assume that similar accuracy could be achieved for furcation defects as for infraosseous defects. Another limitation of the current study is that it only assessed the segmentation of the mandible, not the maxilla. Although SA segmentation is generally accepted as the gold standard for assessing the trueness of DL‐based CBCT segmentations, comparisons with intraoperative direct measurements could further validate the clinical implementation of the current process.

Future research should aim to refine the DL model to address site‐specific variations, particularly in the buccal aspect. Additionally, the model's performance should be investigated across a wider range of defect morphologies, including furcation defects and the periodontal bone topography in the upper jaw, to further expand its utility in periodontal practice. The incorporation of intraoral scans is another future research avenue to enable not just hard tissue defects, but also overlaying soft tissue conditions, to be visualized.

## 5 CONCLUSIONS

The utilized SegResNet‐based DL model demonstrates promising accuracy in the segmentation of infraosseous periodontal defects as per linear measurements. The strong correlations between the INTRA, WIDTH, ANGLE, and volume measurements suggest the reliable capture of defect characteristics. Although measurements in some areas revealed greater differences between the DL and SA segmentations, these were subtle and may not influence clinical treatment planning. These findings support that virtual 3D models obtained via DL‐based CBCT segmentation visualize infraosseous defects reliably and can be valuable tools in periodontal diagnosis and treatment planning.

## AUTHOR CONTRIBUTIONS

*Conceptualization*: Daniel Palkovics, Balint Molnar, Csaba Pinter, David García‐Mato, Andres Diaz‐Pinto, Attila Tanacs. *Writing—original draft*: Daniel Palkovics, Andrea Dobos. *Writing—review and editing*: Balint Molnar, Peter Windisch, Christoph A. Ramseier. *Methodology*: Daniel Palkovics, Csaba Pinter, David García‐Mato, Andres Diaz‐Pinto, Attila Tanacs, Andrea Dobos, Christoph A. Ramseier. *Resources and supervision*: Peter Windisch, Christoph A. Ramseier. *Validation*: Daniel Palkovics, Csaba Pinter. *Software*: Csaba Pinter, David García‐Mato, Andres Diaz‐Pinto, Attila Tanacs. *Investigation, formal analysis, visualization*: Daniel Palkovics.

## CONFLICT OF INTEREST STATEMENT

Andres Diaz‐Pinto, Attila Tanacs, Andrea Dobos, and Christoph A. Ramseier report no conflicts of interest. Daniel Palkovics, Peter Windisch, Balint Molnar, Csaba Pinter, and David García‐Mato are/were founders of the Company Dent.AI 3D guide that developed the utilized SegResNet‐based DL segmentation model. However, the project was entirely self‐funded; no outside funding or support was received for the development of the DL model nor for the preparation of the manuscript.

## Data Availability

The data that support the findings of this study are available from the corresponding author upon reasonable request.
